# Characterization of a panel of monoclonal antibodies recognizing specific epitopes on GFAP

**DOI:** 10.1371/journal.pone.0180694

**Published:** 2017-07-10

**Authors:** Ni-Hsuan Lin, Albee Messing, Ming-Der Perng

**Affiliations:** 1 Institute of Molecular Medicine, College of Life Sciences, National Tsing Hua University, Hsinchu, Taiwan; 2 Waisman Center, University of Wisconsin-Madison, Madison, Wisconsin, United States of America; 3 Department of Comparative Biosciences, University of Wisconsin-Madison, Madison, Wisconsin, United States of America; Medizinische Fakultat der RWTH Aachen, GERMANY

## Abstract

Alexander disease (AxD) is a neurodegenerative disease caused by heterozygous mutations in the GFAP gene, which encodes the major intermediate filament protein of astrocytes. This disease is characterized by the accumulation of cytoplasmic protein aggregates, known as Rosenthal fibers. Antibodies specific to GFAP could provide invaluable tools to facilitate studies of the normal biology of GFAP and to elucidate the pathologic role of this IF protein in disease. While a large number of antibodies to GFAP are available, few if any of them have defined epitopes. Here we described the characterization of a panel of commonly used anti-GFAP antibodies, which recognized epitopes at regions extending across the rod domain of GFAP. We show that all of the antibodies are useful for immunoblotting and immunostaining, and identify a subset that preferentially recognized human GFAP. Using these antibodies, we demonstrate the presence of biochemically modified forms of GFAP in brains of human AxD patients and mouse AxD models. These data suggest that this panel of anti-GFAP antibodies will be useful for studies of animal and cell-based models of AxD and related diseases in which cytoskeletal defects associated with GFAP modifications occur.

## Introduction

Intermediate filaments (IFs) are a highly dynamic cytoskeletal component that provides a structural scaffold and a signaling platform for the organization of the cytoplasm. In humans, at least 70 different IF proteins have been identified [1], many of which are expressed in cell type specific patterns. In astrocytes, GFAP, together with lesser amounts of vimentin [2], nestin [3], and synemin [4], are the major IF proteins that constitute the glial filaments.

Alexander disease (AxD) is a primary astrocyte disease caused by autosomal dominant mutations in the gene encoding GFAP [5]. Clinically, AxD is a fatal leukoencephalopathy that often affects infants and young children. This early-onset Type I form is characterized by symptoms including psychomotor retardation, seizures, and megalencephaly. Milder forms of AxD with variable ages of onset also exist [6]. The Type II form differs markedly in clinical presentation, and patient’s pathology predominantly in the cerebellum, brainstem and cervical spinal cord. Pathologically, AxD is characterized by the presence of ubiquintinated protein inclusions known as Rosenthal fibers (RFs) that are found almost exclusively in astrocytes throughout the central nervous system. In addition to GFAP, RFs have been reported to contain other IF proteins, including vimentin [7, 8], synemin [9], plectin [10], as well as the small stress proteins αB-crystallin and HSP27 [11, 12]. How RFs alter the biochemistry, morphology, and function of astrocytes are not clear. Mouse models created through both transgenic [13, 14] and knock-in [15] approaches showed that simply elevating the level of wild-type GFAP or expressing a mutant form of this protein leads to the formation of RFs. Astrocytes cultured from AxD mice exhibit several biochemical and functional changes, including spontaneous formation of GFAP inclusions, increased caspase activity, decreased cell viability [16] and changes in cell morphology [17]. Cellular models and in vitro studies have provided additional evidence to show that GFAP accumulation increased levels of ubiquitinated GFAP species [18], inhibited proteasome activity [18, 19], and stimulated autophagy [20, 21]. Analyses of GFAP levels and post-translational modifications by antibody-based immunoassays are therefore essential to better understand the pathological sequence of events leading to disease-linked aggregation of glial filaments that occur in the context of this disease.

To achieve this goal, well-characterized anti-GFAP antibodies are needed. Although a large number of antibodies specific to GFAP have been widely available [22–29], few if any of them have defined epitopes. Here we determine the epitopes recognized by a panel of anti-GFAP antibodies and evaluate their use in the analysis of GFAP, with a special focus on biochemically modified forms in samples from human AxD patients and mouse AxD models.

## Materials and methods

### DNA constructs and site-directed mutagenesis

Sets of missense and nonsense mutations were generated by PCR-based site-directed mutagenesis (QuikChange, Stratagene, La Jolla, CA) with use of the human full-length GFAP in the pcDNA3.1(−) vector [30] as a template. All newly generated GFAP constructs were verified by sequencing before use.

### Expression and purification of recombinant proteins

For expression in bacteria, pcDNA 3.1(−) vectors containing either full-length or truncated forms of GFAP were subcloned into the bacterial expression vector pET23b (Merck Millipore, Billerica, MA) using the *XbaI* and *EcoRI* restriction sites. The bacterial expression vectors containing indicated GFAP variants were transformed into *E*. *coli* strain BL21 (DE3) pLysS. GFAP proteins were expressed and purified as previously described [30].

### Caspase cleavage of GFAP in vitro

Purified human recombinant GFAP (1 μg) was diluted in caspase assay buffer (50 mM HEPES, pH 7.2, 50 mM NaCl, 0.1% (w/v) CHAPS, 10 mM EDTA, 10 mM dithiothreitol (DTT) and 5% (v/v) glycerol) in the absence or presence of active recombinant human caspase 3 (BioVision, Milpitas, CA) at a final concentration of 0.25 U/μl. After incubation for 4 hours at 37°C, the cleavage products were analyzed by immunoblotting using indicated anti-GFAP antibodies.

### Peptide synthesis and generation of anti-mouse GFAP antibodies

An immunogen peptide, ASETVVRGLG (amino acids 14–23), which is unique to mouse GFAP (mGFAP), was synthesized, coupled to keyhole limpet hemocyanin and used for immunization (Yao-Hong Biotechnology Inc., Taipei, Taiwan). The rabbit serum providing the highest titer and specificity was subsequently used. The mGFAP-specific antiserum was further purified by affinity chromatography as previously described [31]. For competition assays, peptides corresponding to specific regions of GFAP were synthesized by the Biotechnology Center at the University of Wisconsin-Madison.

### Cell culture

Human adrenal carcinoma SW13 (Vim-) cells (kindly provided by Prof. Michael Brenner, University of Alabama Birmingham), and human adenocarcinoma HeLa cells (kindly provided by Prof. John Svaren, Waisman Center, University of Wisconsin-Madison) were maintained in Dulbecco’s modified Eagle’s medium (DMEM) supplement with 10% (v/v) fetal bovine serum, 2 mM L-glutamine, 100 U/mL penicillin, and 100 μg/ml streptomycin. Unless otherwise stated, all cell culture reagents were purchased from Thermo Fisher Scientific (Waltham, MA). All cells were cultured at 37°C in a humidified incubator of 95% (v/v) air and 5% (v/v) CO_2_.

Primary astrocyte cultures were prepared from 0–2 day old postnatal mice as described previously [16] with minor modifications. The cerebral cortices were dissected in Hank’s balanced salt solution (HBSS) followed by incubation with 0.25% (w/v) trypsin at 37°C for 15 min. After incubating with DNase I (Sigma-Aldrich, St. Louis, MO) for additional 5 minutes, the cortices were mechanically dispersed by triturating with a Pasture pipette. Cells were collected by centrifugation at 1,000 rpm for 5 minutes, followed by resuspension in plating medium (minimal essential medium (MEM) containing 5% (v/v) horse serum, 5% (v/v) fetal bovine serum, and 100 U/ml penicillin and 100 μg/ml streptomycin. After filtration through a 70 μm nylon mesh (Greiner Bio-One, Frickenhausen Germany), cells were seeded onto poly-L-lysine-coated plates or dishes at 2.6 x 10^4^ cells/cm^2^. Cells were cultured for 8–10 days with medium change every 3–4 days.

### Transient transfection and immunofluorescence microscopy

For transient transfection assays, cells grown on 13 mm coverslips at a density of 50–60% confluency were transfected with indicated GFAP constructs using the TransIT-LT1 transfection reagent (Mirus Bio, Madison, WI) according to the manufacturer’s protocol. At 48 hours after transfection, cells were processed for immunofluorescence microscopy as described previously [32]. The primary antibodies used in this study were listed in Table 1. Slides were observed using a LSM 510 confocal laser scanning microscope (Carl Zeiss, Jena, Germany) with a 40× (0.75 NA) Neofluar or 63× (1.40 NA) Apochromat objective lenses. Images were collected in Multitrack mode by LSM510 software taking 1.0 μm optical sections and processed for figures using Adobe^®^ Photoshop CS 6 (Adobe System, San Jose, CA).

**Table 1:** List of primary antibodies used in this study.

Antibody	Host animals	Clone no.	Source	Dilutions	
				IB	IF
GFAP	Rat	2.2B10	[27]	1:5,000	1:400
GFAP	Mouse	SMI-21	BioLegend	1:5,000	1:400
GFAP	Mouse	SMI-23	BioLegend	1:5,000	1:400
GFAP	Mouse	SMI-24	BioLegend	1:5,000	1:400
GFAP	Mouse	SMI-25	BioLegend	1:5,000	1:400
GFAP	Mouse	6F2	Dako	1:1,000	1:100
GFAP	Rabbit		Dako	1:10,000	1:1,000
β-actin	Mouse	AC-15	Novus	1:5,000	
GAPDH	Mouse	1D4	Novus	1:5,000	
αB-cry	Mouse		Enzo	1:5,000	
Ubiquitin	Mouse	P4D1	CST	1:5,000	

IB: immunoblotting; IF: immunofluorescence; CST: Cell Signaling Technology

*This antibody was used as an anti-panGFAP antibody

### Preparation of total cell lysates and immunoblotting

To prepare total cell lysates, transfected cells were lysed on ice in SDS sample buffer (10% glycerol, 2% SDS, 100 mM DTT, 50 mM Tris, pH 6.8) supplemented with cocktails of phosphatase (Clontech) and protease (Roche) inhibitors. Samples were sonicated and heated up at 95°C for 5 minutes prior to immunoblotting analysis.

Immunoblotting was performed using the wet electrophoretic transfer system (Biorad, Hercules, CA) as described previously [32]. Primary antibodies used in this study were anti-GFAP antibodies (Table 1), mouse monoclonal anti-β-actin (AC-15, Novus Biologicals, Littleton, CO), anti-GAPDH (1D4, Novus Biologicals), anti-αB-crystallin (Enzo Life Sciences, Farmingdale, NY), and anti—ubiquitin (P4D1, Cell Signaling Technology, Danvers, MA). Secondary antibodies used in this study were horseradish peroxidase (HRP)-conjugated goat anti-mouse, anti-rabbit or anti-rat (Jackson ImmunoResearch Laboratories, West Grove, PA). Antibody labeling was detected by enhanced chemiluminescence (SuperSignal West Pico Substrate; Thermo Fisher Scientific) with use of a luminescent image analyzer (LAS 4000; GE Healthcare). The strength of signals was quantified using the ImageQuant TL 7.0 software (IQTL, GE Healthcare).

### Human brain samples

Clinical and genetic details of human brain tissues are presented in Table 2. Some tissues were obtained from the NICHD Brain and Tissue Bank for Developmental Disorders at the University of Maryland, Baltimore, MD (supported by NIH Contract #HHSN275200900011C, Ref. No. N01-HD-9-001).

**Table 2:** Clinical and genetic details of Alexander disease patient samples analyzed by immunoblotting.

Case	Disease	Age at onset	Age at death	GFAP mutation	Sex
Control 1	Cardiac arrhythmia	-	14 years	-	M
Control 2	Cardiovascular disease	-	50 years	-	F
Control 3	Normal	-	42 years	-	M
Control 4	Normal (metastatic adenocarcinoma)	-	51 years	-	F
Control 5	Normal (liver failure)	-	46 years	-	F
AxD 1	Infantile-onset/type I	3 months	1 year	R239H	M
AxD 2	Infantile-onset/type I	13 months	6 years	R239C	M
AxD 3	Juvenile-onset/type II	3.5 years	22 years	L359V	M
AxD 4	Adult-onset/type II	37 years	42 years	D417A	F
AxD 5	Adult-onset/type II	25 years	50 years	S247P	F

Sample processing was performed by sequential extraction as described previously [33]. Briefly, frontal or temporal white matter tissues were thawed on ice and dounce homogenized in high salt (HS) buffer (50 mM Tris-HCl, 750 mM NaCl, 1% (v/v) Triton X-100, 5 mM EDTA, 10 mM NaF, pH 7.4). All buffers were supplemented with 1 mM PMSF and cocktails of phosphatase (Clontech) and protease (Roche) inhibitors. Samples were centrifuged at 100,000× *g* for 30 minutes at 4°C and the supernatants were taken as the HS-soluble fraction. Pellets were extracted in HS buffer with 1% (v/v) Triton X-100 and centrifuged at 100,000× *g* for 30 minutes at 4°C. An additional step with homogenization in HS buffer containing 0.85 M sucrose followed by centrifugation was performed after the Triton X-100 buffer extraction to float and remove myelin. The supernatant was discarded, and the pellet was sonicated in HS buffer containing 1% (v/v) Sarkosyl (Sigma-Aldrich). Samples were incubated at room temperature with agitation for 30 minutes prior to centrifugation at 100,000× *g* for 30 minutes at 22°C and the supernatant taken as Sarkosyl-soluble fraction. The remaining pellet, representing the Sarkosyl-insoluble fraction, was extracted with SDS sample buffer (10% glycerol, 2% SDS, 100 mM DTT, 50 mM Tris, pH 6.8) and heated up at 95°C for 5 minutes.

### GFAP knock-in and transgenic mice

Knockin mice (GFAP^R236H/+^) heterozygous for the R236H mutation [15] and transgenic mice (GFAP^Tg^) overexpressing human wild-type GFAP [13] were generated as previously described and maintained in the FVB/N background. GFAP wild type (GFAP^+/+^) mice were used as controls. Unless otherwise stated, mice aged at 8–10 weeks were used for experiments. This study was approved by the Institutional Animal Care and Use Committee of the University of Wisconsin-Madison. All animals were cared for and used in accordance with standards set by the Committee.

### Preparation of mouse brain sections and immunofluorescence

To prepare frozen sections, mice were deeply anesthetized with isoflurane and perfused intracardially with phosphate-buffered saline (PBS), followed by fresh 4% (w/v) paraformaldehyde (Sigma-Aldrich) in PBS. Brains were postfixed in 4% (w/v) paraformaldehyde at 4°C overnight and cryoprotected in graded concentrations of sucrose in PBS (5 hours at 10% (w/v), 20 hours at 20% (w/v), and 48 hours at 30% (w/v)). Tissues were embedded in Tissue-Tek OTC compound (Sakura Finetek) and frozen in a dry ice/ethanol bath prior to sectioning at a thickness of 16 μm on a cryostat.

For immunofluorescence, sections were blocked and permeabilized in 5% (v/v) normal donkey serum (Sigma Aldrich) in PBS with 0.25% (v/v) Triton X-100 for 2 hours. Sections were incubated with indicated anti-GFAP antibodies (Table 1) in PBS containing 1% (w/v) BSA and 0.25% (v/v) Triton X-100 at 4°C overnight. After being washed with PBS several times, sections were incubated with Alexa Fluor^®^ 488 (1:500) and Alexa Fluor^®^ 594 (1:500) conjugated donkey anti-mouse, anti-rabbit, or anti-rat secondary antibodies (Thermo Fisher Scientific). Slides were coverslipped using ProLong Gold mounting media with DAPI (Thermo Fisher Scientific) and images taken with a Zeiss LSM510 confocal microscope.

### Preparation of mouse brain lysates

Mouse brain tissues were thawed on ice and then homogenized in radioimmunoprecipitation assay (RIPA) buffer (20 mM, Tris-HCl, pH 7.5, 150 mM NaCl, 1 mM EDTA, 1% (v/v) Triton X-100, 0.5% (w/v) sodium deoxycholate and 0.1% (w/v) SDS) containing Complete^™^ protease inhibitor cocktail and 1% (w/v) Pefabloc SC (both from Roche). Samples were centrifuged at 17,000×*g* for 20 minutes at 4°C and the supernatant taken as the RIPA-soluble fraction. The remaining pellet, representing the RIPA-insoluble fraction, was homogenized in urea buffer (7 M urea, 50 mM Tris-HCl, pH 7.5, 10 mM NaCl, 1 mM EDTA and Complete^™^ Mini protease inhibitor mixture) and centrifuged at 80,000 ×*g* for 30 minutes at 16°C. The supernatant was taken as the urea-soluble fraction. For each fraction, protein concentration was determined using the bicinchoninic acid protein assay (Thermo Fisher Scientific) prior to analysis by immunoblotting.

### Results

**Fig 1:**
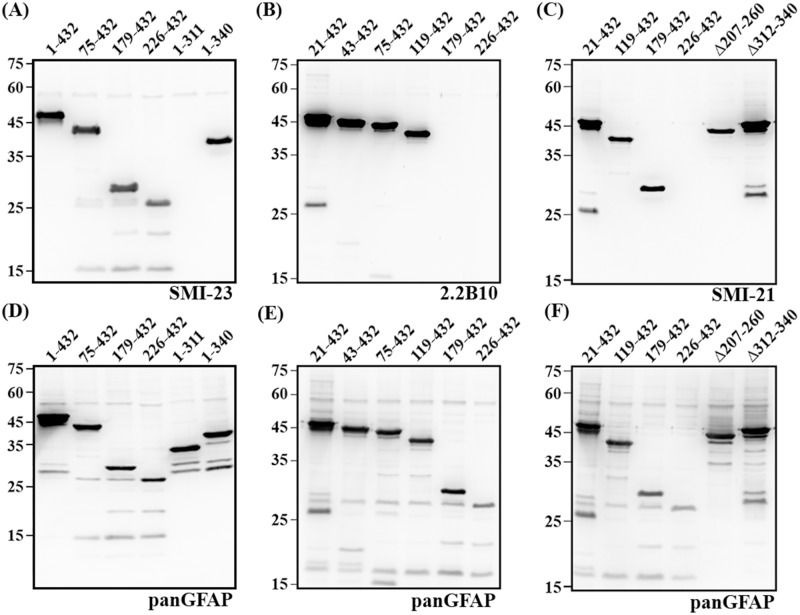
Epitope mapping of anti-GFAP antibodies. HeLa cells were transfected with indicated GFAP constructs for 48 hours. Total cell lysates were prepared and analyzed by immunoblotting with anti-GFAP antibodies as indicated at the bottom of each blot. Representative images showed the immunoblotting pattern for SMI-23 (A), 2.2B_10_ (B) and SMI-21 (C) GFAP antibodies. Immunoblots were also probed with the anti-panGFAP antibody to reveal transfected proteins (D-F). The ability of GFAP antibodies to detect various GFAP proteins is summarized in Table 3.

To determine the epitopes recognized by a panel of pre-existing monoclonal anti-GFAP antibodies, we developed a series of vectors for expression in HeLa cells, a human cervix epitheloid carcinoma cell line that has no endogenous GFAP. Cells transfected with either full-length GFAP or its truncated variants generated proteins of the expected size ranged between 25 and 50 kDa. Immunoblotting showed that although the SMI-23 antibody (Fig 1A) did not react with the amino acids 1–312 of GFAP, it did bind to GFAP residues 1–340, suggesting that the epitope for this antibody lies between amino acid residues 312 and 340 of GFAP. Similar immunoreactive profiles were obtained for other anti-GFAP antibodies, including SMI-24, -25 and 6F2 (S1 Fig), indicating that these antibodies recognized the same or spatially close epitopes on GFAP. Since the 2.2B_10_ antibody recognized all C-terminal truncated GFAP variants, the epitope for this antibody was further defined using N-terminal truncated variants. This antibody recognized all N-terminal deletion mutants, except for the GFAP 179–432 (Fig 1B), indicating that the epitope was contained within amino acids 119–178 of GFAP. These results are summarized in Table 3.

**Table 3:** Summary of the characterization of anti-GFAP antibodies.

GFAP	2.2B_10_	SMI-21	SMI-23	SMI-24	SMI-25	6F2
1–432	+	+	+	+	+	+
1–225	+	+	-	-	-	-
1–311	+	+	+	+	+	+
1–340	+	+	-	-	-	-
1–374	+	+	+	+	+	+
21–432	+	+	+	+	+	+
43–432	+	+	+	+	+	+
75–432	+	+	+	+	+	+
119–432	+	+	+	+	+	+
179–432	-	+	+	+	+	+
226–432	-	-	+	+	+	+
Δ179–206	+	-	+	+	+	+
Δ207–260	+	+	+	+	+	+
Δ312–340	+	+	-	-	-	-
Epitope (aa)	119–178	179–206	312–340	312–340	312–340	312–340
Domain	1B	1B	2B	2B	2B	2B

Truncated GFAP with N-terminal, C-terminal or internal deletions were probed with the indicated anti-GFAP antibodies. The ability of these antibodies to detect truncated forms of GFAP is summarized. (+), Immunopositive; (-), immunonegative. According to the reactivity with different deletion mutants, these antibodies were shown to have their putative epitopes mapped to either N- or C-terminal portion of GFAP. aa, amino acid; Δ, deletion.

**Fig 2:**
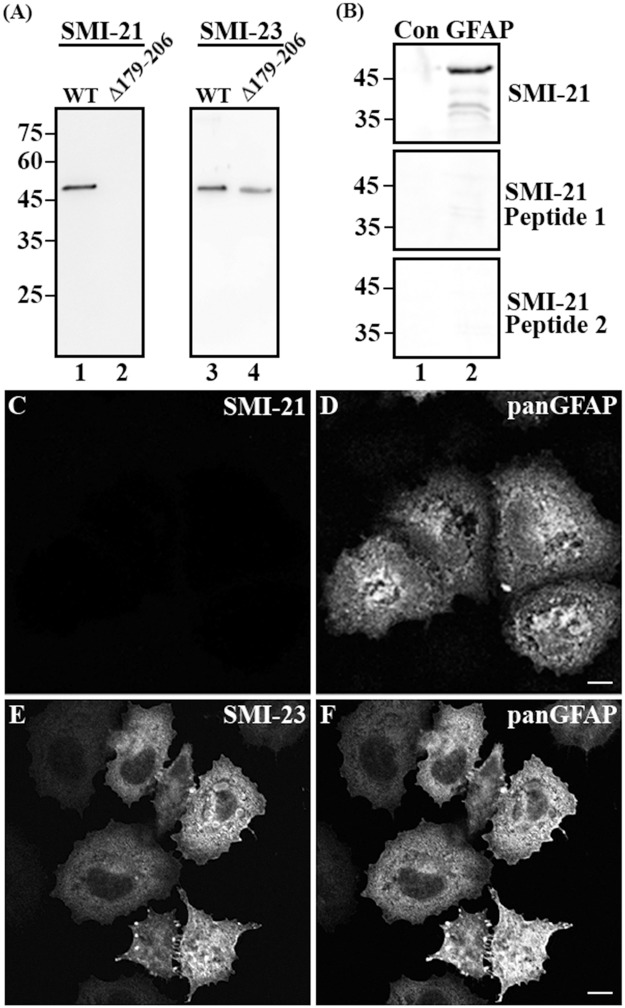
Fine mapping of the SMI-21 antibody epitope on GFAP. (A) Purified recombinant full-length (A, lane 1) and Δ179–206 GFAP (A, lane 2) were probed with either SMI-21 or SMI-23 antibody. The SMI-21 antibody was unable to recognize the GFAP with its putative epitope being removed (A, lane 2). (B) Total lysates prepared from SW13 (Vim-) cells (B, lane 1) expressing human full-length GFAP (B, lane 2) was probed with SMI-21 antibody in the absence (top panel) or presence of peptide 1 corresponding to GFAP residues 186–194 (middle panel), or peptide 2 corresponding to GFAP residues 196–206 (bottom panel). Approximate molecular weight markers (in kDa) were shown on the left. (C-F) SW13 (Vim-) cells transfected with Δ179-206GFAP were immunostained with either SMI-21 (C) or SMI-23 (E) antibody and counterstained with the anti-panGFAP antibody (D and F) to reveal transfected cells. Note that Δ179-206GFAP was readily detected by the SMI-23 antibody (E), but not the SMI-21 antibody (C). Bar = 10 μm.

The SMI-21 antibody has been used as a human GFAP-specific antibody [13, 30, 31, 34, 35], but the location of its epitope remains unknown. Immunoblotting revealed that this antibody recognized all truncated variants tested (Table 3), including 179–432 GFAP (Fig 1C, lane 3) and Δ207–260 GFAP (Fig 1C, lane 5). However, it failed to react with the C-terminal half of GFAP corresponding to amino acids 226–432 (Fig 1C, lane 4). These results indicate that the antibody epitope might be located between amino acids 178 and 207. Removing the putative epitope by generating an internal deletion spanning amino acids 179–206 prevented the antibody from detecting this truncated protein (Fig 2A, lane 2), Peptide competition assays showed that the reactivity of SMI-21 antibody was abolished when pre-incubated with peptides spanning amino acids 186–194 (Fig 2B, Peptide 1) and 196–206 (Fig 2B, Peptide 2), demonstrating that the epitope of the SMI-21 was indeed contained within this region. Immunocytochemistry confirmed that the SMI-21 antibody was incapable of detecting the truncated GFAP missing amino acids 179–206 when transiently expressed in SW13 (vim-) cells (Fig 2C).

**Fig 3:**
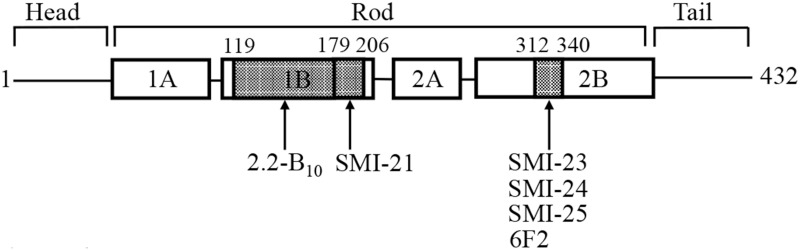
Schematic representation of GFAP domains containing epitopes recognized by the GFAP antibodies. GFAP comprises a central α-helical rod, flanked by the N-terminal head and C-terminal tail domains (denoted by blackbars). Within the central rod domain, the heptad repeat-containing segments (denoted by boxes) are separated by short linker sequences (denoted by black bars). The GFAP domains containing the epitopes (dotted areas) recognized by indicated GFAP antibodies were shown (Table 3). The putative amino acid sequences included in these epitopes were indicated on top of the diagram.

Taken together, we have identified two antibodies (SMI-21 and 2.2B_10_) that recognized specific epitopes in the subdomain 1B of GFAP, and four antibodies (SMI-22, -23, -24 and 6F2) have an epitope located in the subdomain 2B of GFAP (Fig 3).

### GFAP and its biochemically modified forms in the insoluble fraction of AxD brain samples

**Fig 4:**
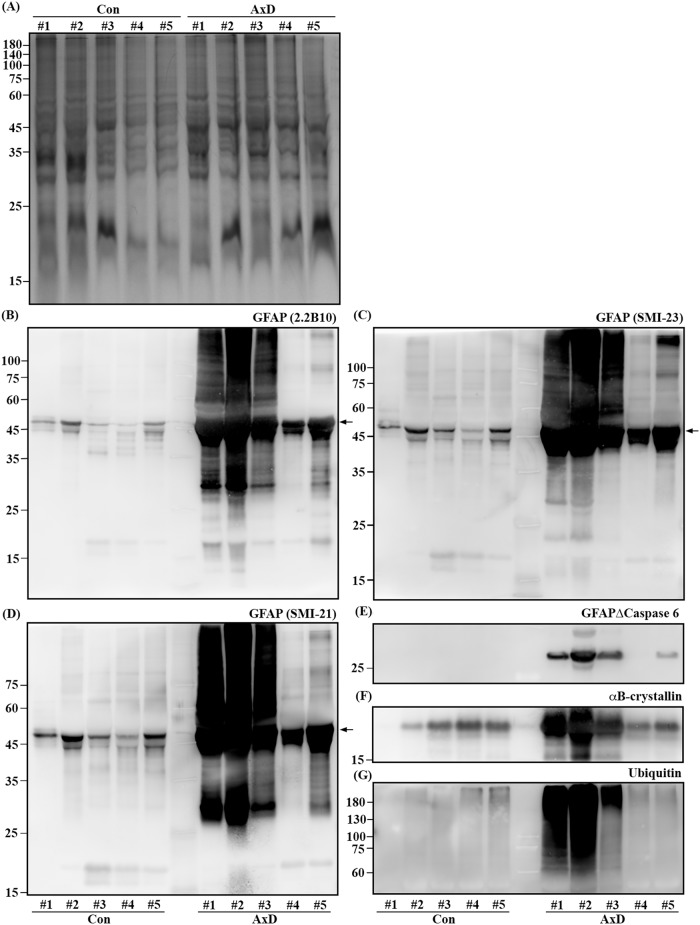
GFAP was ubiquitinated and truncated in brain samples of AxD patients. Proteins from temporal or frontal white matter of five AxD patients (AxD, #1–5) and five non-AxD controls (Con, #1–5) were sequentially extracted with buffers of increasing strength. Sarkosyl-insoluble fractions were analyzed by Coomassie blue staining (A), followed by immunoblotting with 2.2B_10_ (B), SMI-23 (C), and SMI-21 (D) anti-GFAP antibodies. Increased levels of full-length GFAP (B-D, arrows) were consistently observed in AxD brain samples when compared with non-AxD controls. Both 2.2B_10_ (B) and SMI-21 (D) antibodies also detected GFAP fragments sized between 25 and 35-kDa, one of which was confirmed to be caspase-generated fragment by a caspase cleavage site-specific neoepitope antibody (E). Note that the presence of high molecular weight GFAP smear (B-D) correlated with the detection of increased levels of αB-crystallin (F) and ubiquitin (G) in AxD samples with the highest insoluble GFAP levels (AxD, #1–3). Approximate molecular weight markers (in kDa) were shown on the left. Uncropped images of blots (E-G) were shown in S2 Fig.

We then evaluated these anti-GFAP antibodies for their utility in immunoblotting in human brain samples (Table 2), which have been used in a previous study to analyze astrocytic TAR DNA-binding protein 43 (TDP43) in AxD [33]. Temporal or frontal white matter from five AxD-confirmed cases including those infantile-onset type I, juvenile-onset type II, and adult-onset type II diseases were analyzed, along with samples prepared from frontal white matter of human control cases without neurological diseases (non-neurological controls). Human brain tissues were sequentially extracted in buffers with increasing strength and analyzed for levels of total as well as biochemically modified forms of GFAP in the sarkosyl-insoluble but SDS-soluble fractions. Immunoblotting analysis using this panel of anti-GFAP antibodies revealed a general increase in total GFAP levels in all AxD samples examined compared to non-neurological controls (Fig 4B–4D), which was consistent with previous studies [7, 18, 21, 33]. A Coomassie blue stained gel showed similar protein loading for each lane (Fig 4A). Although all antibodies recognized full-length GFAP, both 2.2B_10_ and SMI-21 antibodies also detected 25–35 kDa degradation products in the two infantile-onset type I AxD (AxD#1 and AxD#2) and one juvenile-onset type II (AxD#3) cases. One of the adult-onset type II AxD cases (AxD#5) also had detectable levels of the 25–35 kDa GFAP fragments, which were not detected in another adult-onset type II AxD case (AxD#4) or in any non-neurological controls. In contrast, the SMI-23 and 6F2 antibodies showed a limited immunoreactivity for these degradation products, suggesting that they were N-terminal GFAP fragments. One of these GFAP fragments was likely generated by caspase 6 cleavage, based on its immunoreactivity with a caspase cleavage site-specific neoepitope antibody (Fig 4E) that we had characterized and validated in a previous study [31]. In addition to intact GFAP, all anti-GFAP antibodies also detected high molecular weight smear of GFAP in the three youngest AxD patients (Fig 4B–4D, AxD, #1–3). This correlated with the detection of increased accumulation of αB-crystallin (Fig 4F) and ubiquitin (Fig 4G), suggesting that truncated forms of GFAP associated with the increased AxD pathology present in the most severely affected patients. Thus, this panel of GFAP antibodies with precisely defined epitopes detected intact GFAP as well as its biochemically modified forms, demonstrating their potential utility in helping to define the full range of GFAP species in human AxD brain tissues.

### Elevated levels of GFAP correlated with the detection of degradation products in AxD mouse models

Using these antibodies, we next examined the expression levels and degradation patterns of GFAP in two types of AxD models. While GFAP^*Tg*^ transgenic mice were engineered to constitutively overexpress human wild type GFAP [13], GFAP^*+/R236H*^ knockin mice carry a R236H point mutation in the mouse *Gfap* [15] that is homologous to the common R239H mutation in the human AxD. Both lines exhibit key features of human AxD including increased GFAP levels and widespread deposition of Rosenthal fibers, with GFAP^*Tg*^ mice being the more severely affected of the two models [15].

**Fig 5:**
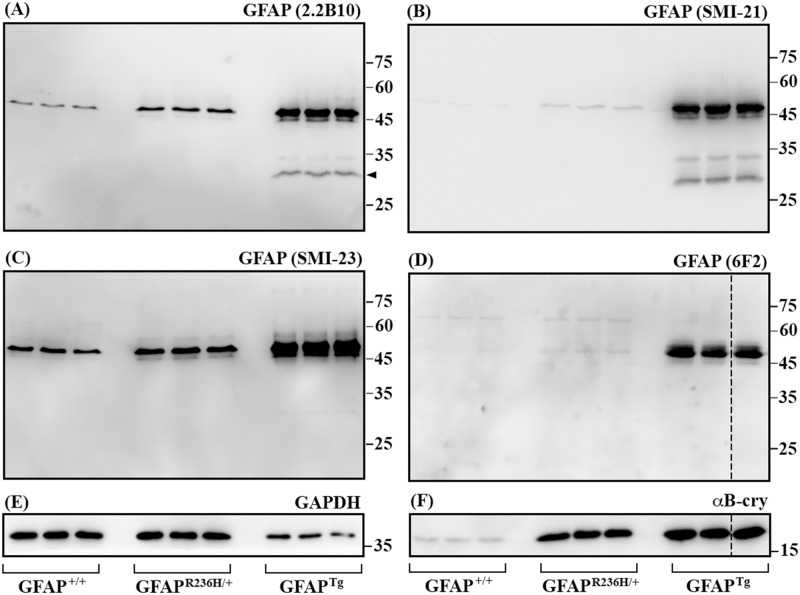
Analysis of GFAP in mouse models of AxD by immunoblotting. Urea-soluble fractions extracted from wild type (GFAP^+/+^), R236H mutant knockin (GFAP^*R236H/+*^) and human wild type GFAP transgenic (GFAP^*Tg*^) mice (n = 3 for each genotype) were analyzed by immunoblotting with indicated anti-GFAP antibodies (A-D). Immunoblots were also probed with antibodies specific to αB-crystallin (F) and GAPDH (E), which was used as a loading control. Representative images were shown, with the antibody used for immunoblotting was indicated above each panel. Approximate molecular weight markers (in kDa) were shown on the right. Dashed lines indicated lanes that samples run on the same gel but were noncontiguous (D and F). Notice that although all anti-GFAP antibodies tested recognized full-length GFAP (A-D), both 2.2B_10_ (A) and SMI-21 (B) antibodies also detected GFAP degradation products sized between 25 and 35 kDa in GFAP^*Tg*^ mice. The level of αB-crystallin (F) was increased in both GFAP^*R236H/+*^ and GFAP^Tg^ mice compared to wild type controls (GFAP^+/+^).

**Fig 6:**
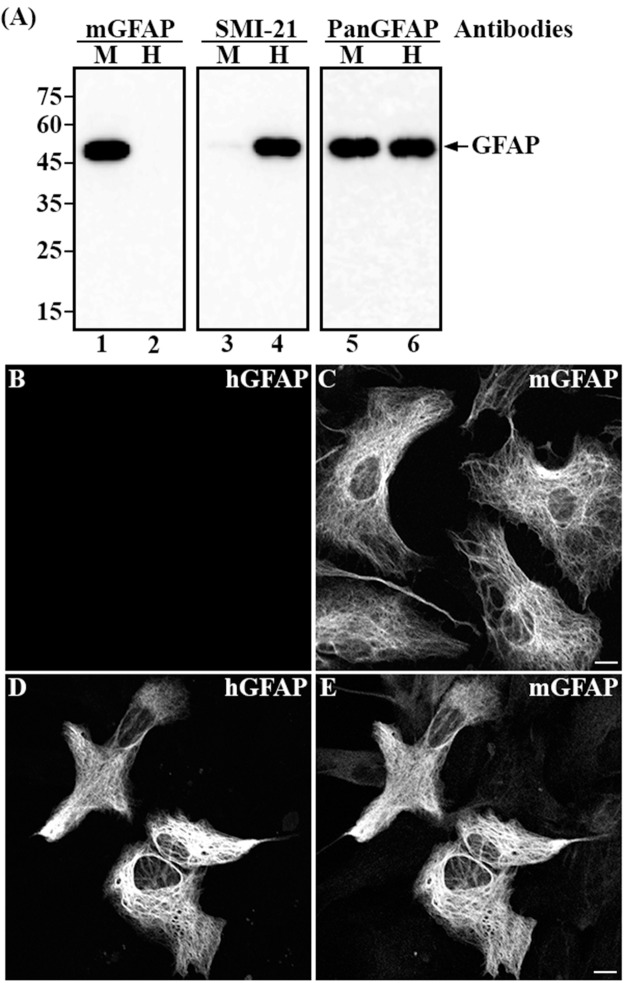
Characterization of polyclonal antibodies specific to mouse GFAP. (A) Purified recombinant mouse (A, lanes 1, 3 and 5, labeled M) and human (A, lanes 2, 4 and 6, labeled H) GFAPs were probed with anti-mouse GFAP (A, lanes 1 and 2), anti-human GFAP (SMI-21) (A, lanes 3 and 4) and anti-panGFAP (A, lanes 5 and 6) antibodies. Notice that the anti-mouse GFAP (A, lane 1) and SMI-21 (A, lane 4) antibodies recognized mouse and human GFAP, respectively, confirming the specificity of these antibodies. The anti-panGFAP antibody, however, recognized both mouse and human GFAPs (A, lanes 5 and 6). Approximate molecular weight markers (in kDa) were shown on the left. Primary mouse astrocytes (B and C) were transiently transfected with human wild type GFAP (D and E). At 48 hours after transfection, cells were immunostained with SMI-21 (B and D) and anti-mouse GFAP antibodies (C and E). When expressed in mouse primary astrocytes, human wild-type GFAP formed filamentous networks (D) that colocalized with the endogenous mouse GFAP (E). Bar = 10 μm.

Like other intermediate filament family members, GFAP normally exists in dynamic equilibrium between Triton X soluble and insoluble pools. Using the RIPA extraction buffer, GFAP was fractionated primarily into a RIPA-insoluble but urea-soluble fraction [31, 33]. Immunoblotting of the urea-soluble fraction revealed that both SMI-21 (Fig 5B) and 6F2 (Fig 5D) antibodies detected GFAP in GFAP^*Tg*^ transgenic mice but not in GFAP^*+/R236H*^ knockin and GFAP^*+/+*^ control mice, suggesting that these antibodies specifically recognized human GFAP (hGFAP) but not mouse GFAP (mGFAP). In contrast, 2.2B_10_ (Fig 5A) and SMI-23 (Fig 5C) antibodies detected GFAP in mice of all genotypes, indicating that they reacted with both mGFAP and hGFAP. Quantitative analysis of the intact GFAP using the 2.2B_10_ antibody showed a 3.2-fold (3.2±0.3, n = 3) increase in GFAP^*R236H/+*^ mice and 26-fold (25.9±1.8, n = 3) increase in GFAP^*Tg*^ mice compared to wild type (GFAP^+/+^) controls (Fig 5A). In addition to intact GFAP, the 2.2B_10_ antibody also detected degradation products with sizes ranging between 25 and 35 kDa in the GFAP^*Tg*^ mice. The average level of the major GFAP fragment (Fig 5A, arrow) was measured to be 16.2%±0.5% (n = 3) of the full-length protein. These findings suggest that elevated GFAP levels correlate with proteolytic cleavage of GFAP to generate N-terminal fragments as a consistent feature of AxD. The level of αB-crystallin, a component of Rosenthal fibers, was also increased in GFAP^*+/R236H*^ knockin and GFAP^*Tg*^ transgenic mice compared to wild type controls (Fig 5F), consistent with a recent study that αB-crystallin was up-regulated in response to accumulation of GFAP [7].

The human-specific SMI-21 and 6F2 GFAP antibodies would be useful for the study of AxD mice transgenic for hGFAP. To differentiate mouse versus human GFAP in the GFAP^*Tg*^ mice, we sought to develop antibodies that specifically recognized mGFAP but not hGFAP. The immunogenic peptide specific for the mGFAP elicited polyclonal antibodies that specifically recognized mGFAP, as judged by immunoblots of purified recombinant proteins (Fig 6A). Suitability of these antibodies for immunohistochemistry was demonstrated by immunostaining of cultured primary mouse astrocytes (Fig 6B and 6C) transiently transfected with human wild-type GFAP (Fig 6D and 6E).

**Fig 7:**
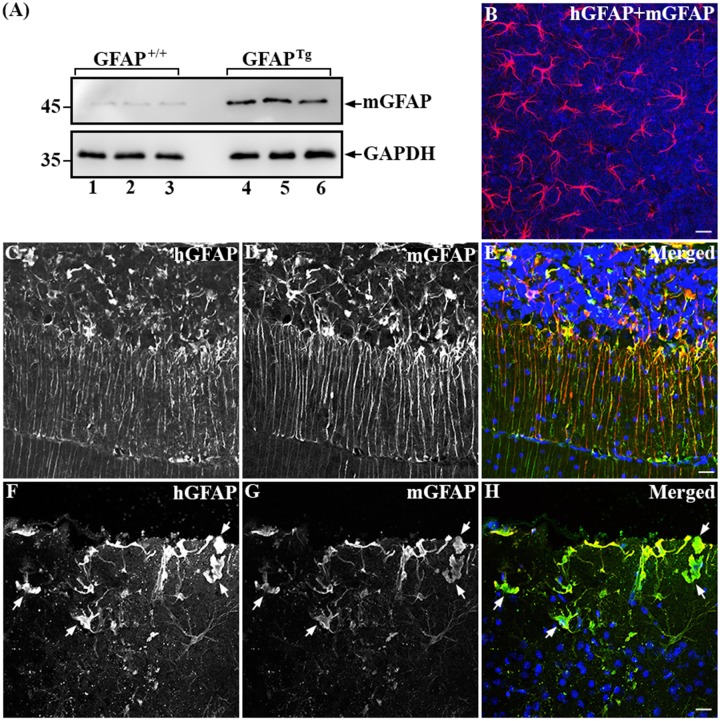
Upregulation and accumulation of the endogenous mouse GFAP in the GFAP^*Tg*^ mice. Urea-soluble fractions prepared from whole brains of GFAP^+/+^ wild type (A, lanes 1–3) and GFAP^*Tg*^ (A, lanes 4–6) mice were analyzed by immunoblotting using the anti-mouse GFAP antibody. Immunoblots probed with anti-GAPDH antibody was used as a loading control. Note that the endogenous mouse GFAP was significantly increased in the GFAP^*Tg*^ mice (A, lanes 4–6) compared to wild type controls (A, lanes 1–3). Approximate molecular weight markers (in kDa) were shown on the left. The distributions of human GFAP in relation to the endogenous mouse GFAP in brain sections of wild type (GFAP^+/+^, B) and GFAP^*Tg*^ (C-H) mice were visualized by double-label immunofluorescence microscopy using anti-mouse GFAP and SMI-21 antibodies. The immunofluorescence for human GFAP was in the green channel (C and F), whereas the counterstaining for mouse GFAP was in the red channel (D and G). Merged images showed the superimposition of both the green and red signals, with overlapping area appearing yellow (B, E, and H). Nuclei were revealed by staining with DAPI (B, E, and H). In wild type mice, normal appearance of GFAP in astrocytes was readily stained with the anti-mouse GFAP antibody (B), with very little staining with the SMI-21 antibody. Conversely, GFAP^*Tg*^ mice showed numerous Rosenthal fiber-like GFAP aggregates in the granular cell layer of the cerebellum (C-E) and the pial surface of the cortex (F-H), which were immunopositive for both mouse and human GFAP. Bar, 10 μm.

Using the mGFAP—specific antiserum, we first determined the level of the endogenous mGFAP in the GFAP^*Tg*^ mice. Analysis of the urea-soluble fraction by immunoblotting revealed that the endogenous mGFAP was increased 5.2-fold (5.16±0.97, n = 3) in GFAP^*Tg*^ mice (Fig 7A, lanes 4–6) compared to wild type controls (Fig 7A, lanes 1–3). Such an increase in the mGFAP level in GFAP^*Tg*^ mice might reflect up-regulation of the endogenous mGFAP gene due to astrocyte activation, a prominent feature of the GFAP^*Tg*^ mice [36]. The distribution of hGFAP in relation to the endogenous mGFAP in astrocytes of the GFAP^*Tg*^ mice was visualized by double-label immunofluorescence microscopy using the anti-mGFAP antibody and the SMI-21 antibody. The anti-mGFAP antibody readily stained astrocytes in brain sections of control wild type mice (Fig 7B), but the SMI-21 antibody produced little signal, confirming the immunoblots results that the SMI-21 antibody did not crossreact with mouse GFAP (Fig 6A, lane 3). In GFAP^*Tg*^ mice, however, Rosenthal fiber-like aggregates that were immunopositive for both hGFAP and mGFAP were apparent in the granular cell layer of the cerebellum (Fig 7C–7E) and the pial surface of the cortex (Fig 7F–7H).

## Discussion

### GFAP has some unique and highly immunogenic epitopes

This is the first comprehensive study to map the epitopes for a panel of monoclonal antibodies specific to GFAP, and we also illustrate their application to detect modified forms of GFAP in human AxD patients and mouse AxD models. The epitopes for SMI-23, -24 and -25 were mapped to the subdomain 2 between amino acids 312–340 of GFAP. This region is highly conserved, and is identical between amino acids 319 and 340 in the human and mouse GFAP. Although the epitope of the 6F2 antibody also mapped to the same region, this antibody only recognized human GFAP [37–40], suggesting that it was directed against an epitope that is distinct from that seen by the SMI-23, -24 and -25 antibodies. Within this region, the GFAP sequence differs between human and mouse GFAP at three amino acid residues including D^315^, A^316^ and A^318^ (in the numbering of human GFAP). These amino acid differences therefore may account for the specificity of the 6F2 antibody for the human protein.

The SMI-21 antibody has for many years been used as a human GFAP-specific antibody [13, 30, 31, 34, 35], and we have mapped the epitope of this antibody to the amino acids 179–206 corresponding to the 1B subdomain of GFAP. The sequence in this region is identical between human and mouse GFAP except for amino acids I^190^, R^198^ and H^204^, suggesting that these residues are important for antibody binding. The 2.2B_10_ antibody was raised against an immunogen derived from gel-excised bovine GFAP [27] and the epitope of this antibody was mapped to the 1B subdomain of GFAP corresponding to amino acids 119–178. This region is highly conserved among different species, thus explaining the ability of this antibody to recognize both mouse and human GFAP. Additional studies will be required to further map the epitope of the 2.2B_10_ antibody to a smaller region of GFAP. It is important to note, however, that our interpretation of the epitope mapping data is based primarily on the immunoreactivity of these antibodies to the linear sequence of GFAP. We cannot exclude the possibility that some of these antibodies may recognize epitopes in a conformation-dependent manner.

In addition to GFAP and its biochemically modified forms, this panel of GFAP antibodies might be able to recognize different isoforms of GFAP. To date, there are at least 9 spice variants identified in different species, including human, mouse, and rat [41]. The most abundant isoform GFAP-α has 9 exons, which encode a characteristic tripartite domain structure comprising a central α-helical rod domain flanked by non-helical N-terminal “head” and C-terminal “tail” domains (Fig 3). While GFAP-β [42, 43] and GFAP-γ [44] are splicing variants that are likely to have unique sequences in the N-terminal head domain, GFAP-δ [45, 46], GFAP-κ [47], and GFAP-ζ [44] are alternatively spliced isoforms with variable sequences in the C-terminal tail. The ΔEx6, ΔEx7, Δ135 and Δ164 transcripts encode four splicing variants collectively known as GFAP^+1^, which contain frame-shifted C-termini [48] that change the length of the rod domain and the sequence of the tail. Because these different GFAP isoforms mainly differ in the sequences of the head and tail domains without affecting most of the rod, we expect our panel of anti-GFAP antibodies is capable of detecting all GFAP isoforms. For instance, we had previously shown that the SMI-21 antibody recognized both GFAP-α and -δ in cultured astrocytoma cells and in human spinal cord [35]. In addition, the mouse GFAP-specific antibody with its predefined epitope at the N-terminal head is expected to detect most of the mouse GFAP isoforms, except for the GFAP-γ that lacks exon1 leading to a shortened N-terminal sequence [44].

### GFAP antibodies as a tool to monitor GFAP proteolysis

Our data showed that some GFAP antibodies detected truncated forms of GFAP in addition to full-length protein in samples from AxD mice and human patients but not in those from normal controls. Thus the presence of the truncated forms of GFAP in AxD is abnormal and seems to be limited to injury or disease. GFAP is susceptible to proteolysis both in vivo and in vitro. The proteases responsible for GFAP proteolytsis include calpains and caspases. An early study showed that GFAP is cleaved in vitro and in situ by calcium-dependent proteinase [49]. Zhang et al. found that this proteinase could be calpains [50], which cleave GFAP in vitro at N59–A60 and T383–F384 producing a cluster of breakdown products between 38 and 50 kDa [51]. Increase in calpain-generated GFAP fragments was similarly detected in the spinal cord of amyotrophic lateral sclerosis [52] and in experimental brain injury of rodents as well as in cerebrospinal fluid (CSF) of traumatic brain injury patients [53]. However, our data are not compatible with the possibility that the GFAP degradation products in AxD resulted from calpain action, since immunoblotting of both mouse and human samples showed proteolytic fragments between 25 and 35 kDa as the predominant degradation products. Of these, a ~26 kDa band was the most abundant fragment detected in four of the five AxD cases examined. This fragment was confirmed to be caspase 6-generated, as the unmasked neoepitope at VELD^225^ of GFAP was recognized specifically by an epitope-specific antibody [31]. In addition to the 26 kDa fragment, other forms of GFAP degradation products that vary in size and quantity also exist. For instance, a ~30 kDa fragment, when detected by adjusting the sample loading amount, was found to be at much lower levels than the 26 kDa fragment in AxD patients (S3A Fig). This degradation product most likely corresponds to a caspase 3 generated fragment, because it migrated similarly to a degradation product that was detected in cells undergo apoptosis achieved by overexpressing a mutant form of GFAP (S3A Fig) and in vitro by cleavage of purified recombinant GFAP with active caspase 3 (S3B Fig). In support of these data, previous studies found that a 30 kDa GFAP fragment generated by caspase 3 cleavage at a unique DLTD^266^ site was present in reactive astrocytes of the Alzheimer disease brain [54].

Our findings suggest that proteolytic modifications of GFAP might be a common event in the setting of disease. However, it is possible that these fragments normally exist, and that their detection in the context of disease might simply reflect detection threshold that is more easily exceeded when GFAP levels are highly elevated. We do not know whether these truncated forms have any pathological significance in AxD. Nor can we be certain whether all GFAP fragments will play the same role in the pathogenesis. Although our data suggest that the level of GFAP degradation products might correlate with the disease severity, because increased levels of GFAP fragments were found mainly in younger AxD patients, testing of such a hypothesis awaits the availability of more human neuropathological materials from AxD patients.

In addition to caspase and calpain, the proteasome [18, 19] and autophagy [20, 21] pathways are also involved in the degradation of GFAP, which in combination with synthesis help define the turnover of this IF protein. Although considerable evidence supports the idea that GFAP synthesis is regulated at the transcriptional level [55, 56], only a few studies have examined the degradation of GFAP. For instance, kinetic studies on turnover of GFAP in cultures of primary astrocytes showed a half-life ranging from 18 hours to 8 days [57, 58]. In vivo, however, the turnover rate of GFAP is much longer, with reported half-lives of 4–9 weeks [59, 60]. Recently, Moody et al. [61] showed that the turnover half-life of GFAP in the R236H knockin mice (15.4 ± 0.5 days) was much shorter compared to wild-type littermates (27.5 ± 1.6 days), suggesting that the presence of mutant GFAP increases not only synthesis but also degradation. The mechanisms that target GFAP for different degradation pathways and the significance of these proteolytic events have yet to be determined. Nevertheless, the GFAP antibodies described here will be useful in defining the links that connect proteolytic processing of GFAP to disease-linked aggregation of glial filaments.

### GFAP fragment could serve as a biomarker for AxD

Given the wide spectrum of clinical presentations and courses for AxD, there is an urgent need to identify and evaluate AxD-specific biomarkers that could serve as a convenient means to monitor the progression of disease and the response to treatment. Unfortunately, well-characterized and validated biomarkers specific for AxD do not exist at present. We consider that GFAP itself could be a biomarker because elevation of total GFAP levels is a key factor in the pathogenesis and GFAP levels are consistently elevated in CSF and blood of patients with AxD [62, 63].

Because of low absolute levels, analysis of GFAP in biofluids requires sensitive immunoassays, such as enzyme-linked immunosorbent assay (ELISA). Although ELISA has been shown to reliably measure GFAP levels in CSF and plasma [64], current versions lack the potential for the detection of post-translational modifications of GFAP [65–67], nor do they distinguish between full-length protein and forms that are truncated through degradation. Although a recent study showed that GFAP could be detected in CSF and serum from patients with AxD [62], the precise forms of GFAP that were detected was not identified. Given that GFAP degradation products were detected in four out of five human AxD samples examined here (Fig 4), GFAP in the biofluids of AxD patients are likely to be, in part, in the form of degradation products.

To utilize GFAP as an effective biomarker applicable for AxD, it is likely that truncated forms of GFAP will need to be quantified separately from intact protein. Although detection of GFAP degradation products alone seems difficult, an antibody has been reported to be specific enough to potentially achieve this goal. Using a proprietary antibody, GFAP degradation products can be detected in CSF in patients with traumatic brain injury by ELISA [68], although the epitope that distinguishes truncated versus intact forms of GFAP has yet to be revealed. Since increasing evidence suggests that GFAP degradation products might serve as biofluid-based markers for numerous neurological conditions [64, 69], further efforts should be made to find the disease-specific degradation pattern of GFAP unique for AxD. The detection of disease-specific GFAP fragments either alone or in combination with other biomarkers [70] will extend the utility of anti-GFAP antibodies for future patient-oriented research as well as experimental studies on animal- and cell-based models of AxD and possibly other related diseases where GFAP proteolysis is a prominent feature.

## Supporting information

S1 Fig Epitope mapping of anti-GFAP antibodies. Cell lysates from transfected HeLa cells were prepared as described in the Fig 1. Samples were analyzed by immunoblotting using SMI-24 (A), SMI-25 (B), and 6F2 (C) anti-GFAP antibodies. Corresponding blots were probed with anti-panGFAP antibodies to reveal GFAP expressed in transfected cells (D-F).
(PDF)

S2 Fig Full-length images of blots from Fig 4. Samples prepared from five AxD patients (AxD, #1–5) and five non-neurological controls (Con, #1–5) were analyzed by immunoblotting using antibodies to a neoepitope at N-terminal GFAP ending with VELD^225^ (A), αB-crystallin (B) and ubiquitin (C). Molecular mass markers (in kDa) were shown on the left.
(PDF)

S3 Fig Compared of GFAP degradation products in AxD brain samples and in HeLa cells expressing GFAP. (A) Immunoblotting analysis of a brain sample from the AxD patient harboring R239H GFAP (AxD#1) by the SMI-21 antibody revealed two proteolytic fragments (lane 1), which were of similar sized as the corresponding fragments generated in HeLa cells transiently transfected with R239H GFAP (lane 2, p30 and p26 indicated by arrows). (B) Purified recombinant R239H GFAP was either untreated (lane 1) or treated with 2.5 U of active caspase 3 (lane 2) for 1 h at 37°C. The reaction products were analyzed by immunoblotting using the polyclonal anti-panGFAP antibodies. Note that GFAP cleaved by active caspase 3 generated two prominent proteolytic fragments, p30 and p20 (B, lane 2, arrows).
(PDF)
