# Supplementary figures and images for: Characterization of a panel of monoclonal antibodies recognizing specific epitopes on GFAP
Source: PLoS One. 2017 Jul 10;12(7):e0180694. doi: 10.1371/journal.pone.0180694 (PMC5503259; doi:10.1371/journal.pone.0180694)

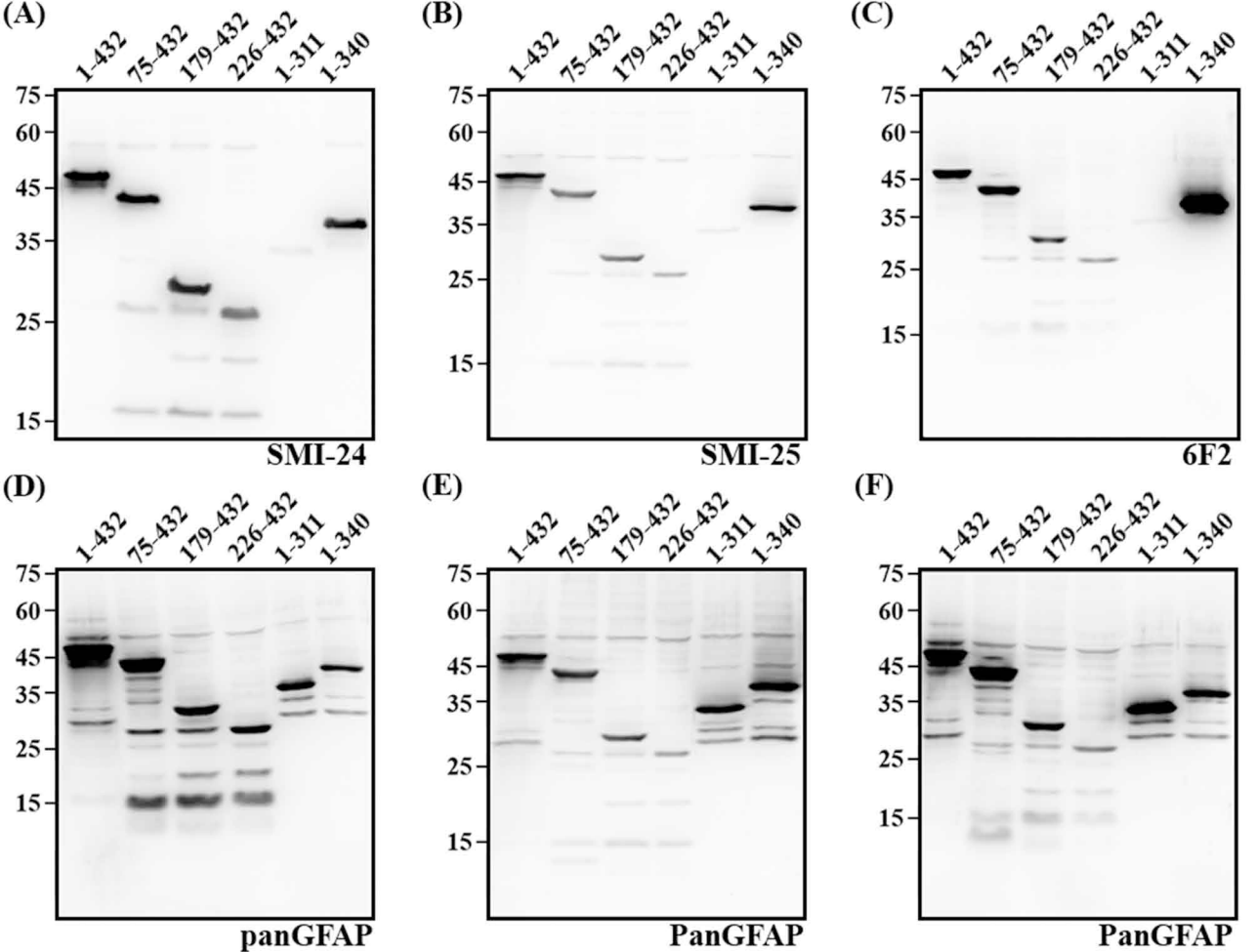

Supplement: S1 Fig — Cell lysates from transfected HeLa cells were prepared as described in the Fig 1. Samples were analyzed by immunoblotting using SMI-24 (A), SMI-25 (B), and 6F2 (C) anti-GFAP antibodies. Corresponding blots were probed with anti-panGFAP antibodies to reveal GFAP expressed in transfected cells (D-F). (PDF) [file pone.0180694.s001.pdf]

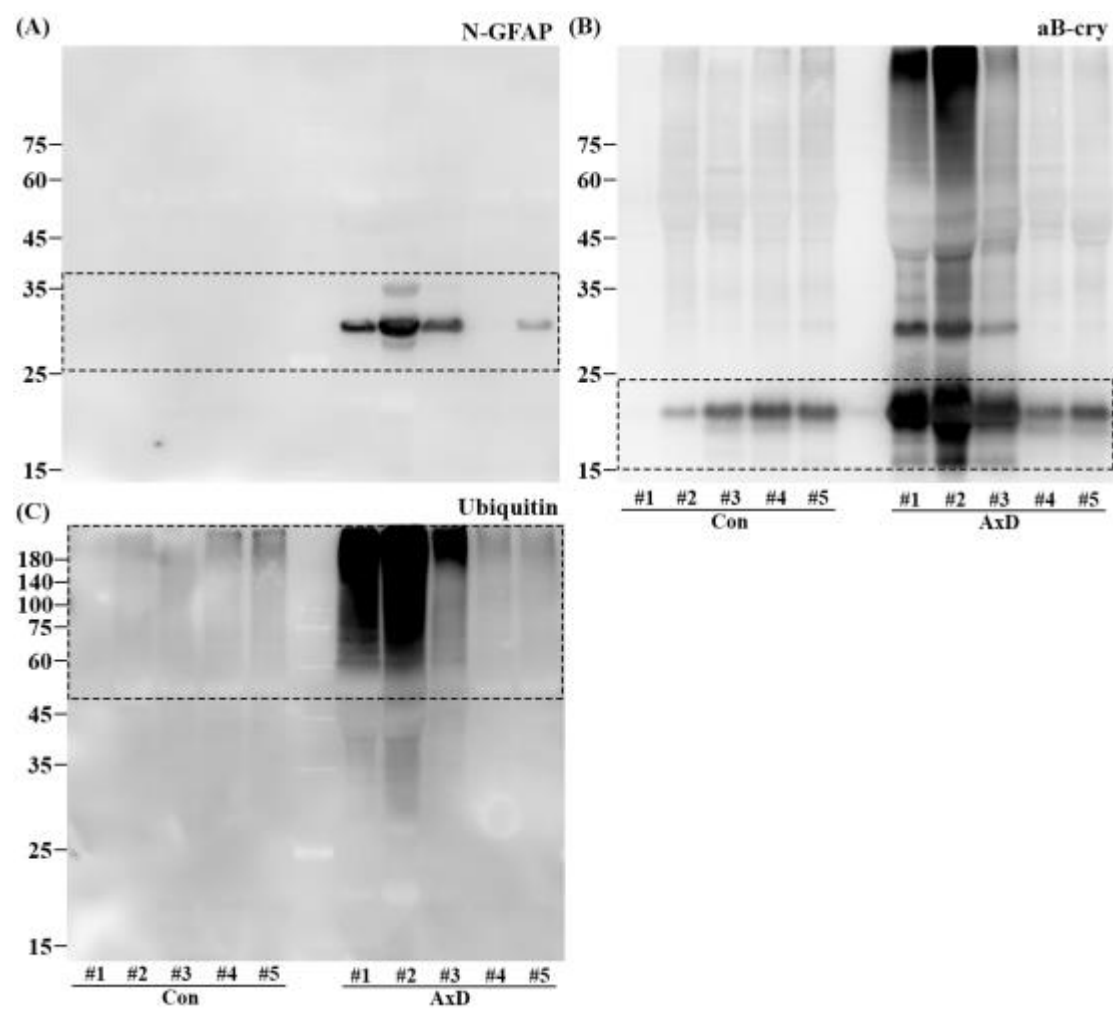

Supplement: S2 Fig — Samples prepared from five AxD patients (AxD, #1–5) and five non-neurological controls (Con, #1–5) were analyzed by immunoblotting using antibodies to a neoepitope at N-terminal GFAP ending with VELD225 (A), αB-crystallin (B) and ubiquitin (C). Molecular mass markers (in kDa) were shown on the left. (PDF) [file pone.0180694.s002.pdf]

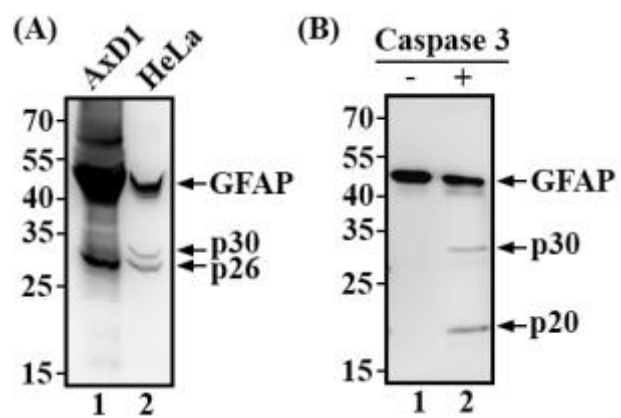

Supplement: S3 Fig — (A) Immunoblotting analysis of a brain sample from the AxD patient harboring R239H GFAP (AxD#1) by the SMI-21 antibody revealed two proteolytic fragments (lane 1), which were of similar sized as the corresponding fragments generated in HeLa cells transiently transfected with R239H GFAP (lane 2, p30 and p26 indicated by arrows). (B) Purified recombinant R239H GFAP was either untreated (lane 1) or treated with 2.5 U of active caspase 3 (lane 2) for 1 h at 37°C. The reaction products were analyzed by immunoblotting using the polyclonal anti-panGFAP antibodies. Note that GFAP cleaved by active caspase 3 generated two prominent proteolytic fragments, p30 and p20 (B, lane 2, arrows). (PDF) [file pone.0180694.s003.pdf]
